# CAREGIVERS OF OLDER ADULTS: AN ANALYSIS OF THE PCORI FUNDED RESEARCH PORTFOLIO

**DOI:** 10.1093/geroni/igz038.3145

**Published:** 2019-11-08

**Authors:** Marissa Rurka, Carly P Khan, Rachel Witsaman, Steven Susana-Castillo, Sindhura Gummi, Gyasi Moscou-Jackson, Steven B Clauser, Neeraj K Arora

**Affiliations:** 1 Purdue University, Lafayette, Indiana, United States; 2 Patient-Centered Outcomes Research Institute, Washington, District of Columbia, United States; 3 Yale School of Public Health, New Haven, Connecticut, United States; 4 PCORI, District of Columbia, United States; 5 Patient-Centered Outcomes Research Institute, District of Columbia, United States

## Abstract

A family-centered approach to care is vital, and caregivers play an important role in patient-centered care for older adults. This analysis of the Patient-Centered Outcomes Research Institute’s (PCORI) portfolio of clinical comparative effectiveness research (CER) trials explores the extent to which caregiving for older adults is a focus within our funded studies and examines how these studies incorporate interventions and outcomes related to caregivers. Of 116 studies in the portfolio with a caregiving component, only 35 studies focus on caregivers of older adults. Approximately half of these studies (16) were not focused on a specific disease, but rather included older adults with a variety of diseases. Caregivers were the target of a delivered intervention in 18 studies. Among these studies, all but one included caregivers as part of a multicomponent intervention. The most common intervention components were caregiver training (14 studies) and inclusion of caregivers in the delivery of health services, notably coordination of care (17), home visits (9), integrated care (9), multidisciplinary care teams (9), and clinical decision tools (8). Caregiver-focused outcomes were assessed in 26 studies. The most frequently assessed domains include measures of health and well-being (most commonly psychosocial status; n=20), evaluation of care (most commonly satisfaction; n=8), and health behavior (most commonly attitudes; n=6). In general, given stakeholder interest in family-centered research on older adults, future CER research should include caregivers and/or compare interventions focused solely on the unique needs of caregivers of older adults. Inclusion of caregiver-related outcomes should also be promoted.

